# Infection of *Trichinella spiralis* Affects the Reproductive Capacity of ICR/CD-1 Male Mice by Reducing the Urine Pheromone Contents and Sperm Quality

**DOI:** 10.3390/ijms24065731

**Published:** 2023-03-17

**Authors:** Gaojian Li, Tao Zhang, Bin Hu, Shuyi Han, Chen Xiang, Guohui Yuan, Hongxuan He

**Affiliations:** 1 National Research Center for Wildlife-Borne Diseases, Institute of Zoology, Chinese Academy of Sciences, Beijing 100101, China; 2 University of Chinese Academy of Sciences, Beijing 100101, China

**Keywords:** *Trichinella spiralis*, reproductive injury, urine pheromone content, sperm quality, ICR/CD-1 male mice

## Abstract

Female mice can discriminate the urinary odors of male mice due to their olfactory acuity. Parasitic infection or subclinical infection can decrease the odor attractiveness of male mice and finally lead to aversion or avoidance responses in odor selection for female mice. *Trichinella spiralis* is a kind of tissue-parasitizing nematode that causes trichinellosis, a zoonotic parasitic disease that spreads throughout the world. However, the reproductive injury caused by *Trichinella spiralis* infection was not fully revealed. In this study, we explored the effect of *Trichinella spiralis* infection on the reproductive capacity in ICR/CD-1 male mice. We identified eight volatile compounds in urine by GC-MS analysis, and the results indicated that the contents of dimethyl sulfone, Z-7-tetradecen-1-ol, 6-Hydroxy-6-methyl-3-heptanone and (S)-2-sec-butyl-4,5-dihydrothiazole were significantly downregulated after parasitic infection, which might lead to the reduction of attractiveness of male mice urine to females. On the other hand, parasitic infection decreased sperm quality and downregulated the expression levels of *Herc4*, *Ipo11,* and *Mrto4*, and these genes were strongly related to spermatogenesis. In summary, this study revealed that the reproductive injury caused by *Trichinella spiralis* infection in ICR/CD-1 male mice could be associated with a decrease in urine pheromone content and sperm quality.

## 1. Introduction

The relationship between parasitic infection and reproductive injury had been reported [1,2,3,4,5], and most studies focused on the decreased sperm quality caused by parasitic infection. On the other hand, the mate choice of female mice could also be influenced by the parasitic infection of male mice, and females preferentially chose the parasite-free or parasite-resistant males [6]. It should be noted that the odor of male mouse urine has a profound effect on the *Mus musculus* mate choice [7]. Studies had suggested that the change in immune function had an important influence on physiology and mate choice [8]. The attack of infectious pathogens changed the urine odor in male mice, and the females could discriminate the uninfected male mice [9]. However, the decrease in sexual attraction of males to females in small rodents was rarely studied.

Rodent urine contains a class of lipocalin, which are major urinary proteins (MUPs). MUPs play a crucial role in chemical communication as they can bind, concentrate, and slowly release volatile pheromones including 2-sec-butyl-4,5-dihydrothiazole, 6-hydroxy-6-methyl-3-heptanone [10,11], geraniol, farnesene, dihydrofurans, and 3,4-dehydro-exo-brevicomin [12,13], which makes MUPs an important chemical signal source in mate choice. Furthermore, MUPs become reliable signals of the competitiveness of males who have successfully occupied territories [14]. The expression of MUP has some plasticity [15,16,17], and health status can be a major factor as it can influence the energy expenditure for sexual signal production in male mice [18]. MUPs make up about 90% of the total protein content in male mice urine and ensure the controlled release of volatile compounds [19]. Both the concentration and fractional composition of MUPs determine the personality and attractiveness of the male scent marks [20].

*Trichinella spiralis* is a kind of tissue-parasitizing nematode that causes trichinellosis when humans are infected with it, this disease is a zoonotic parasitic disease and spreads widely [21]. *Trichinella spiralis* can infect both humans and animals (e.g., pigs) and approximately 11 million people are chronically infected in the world [22]. The severity of trichinellosis is strongly related to the number of infective larvae [23] and the symptoms can vary from mild to severe [24]. Acute infection shows gastrointestinal symptoms, followed by subsequent inflammatory responses like fever, myalgia, and ocular or facial oedema. Symptoms usually disappear within a few months, but the encysted larvae in muscles can survive for a long time. Furthermore, the infection pattern of *Trichinella spiralis* is changing, with the disease re-emerging in some previously disease-free areas [25]. Studies had reported that the *Trichinella spiralis* infection increased the number of single-chain breaks and alkaline-labile sites in nuclear DNA, and the count of apoptotic cells in the bone marrow and testes in infected male mice [26].

In the current study, we investigated the reproductive injury caused by *Trichinella spiralis* infection in ICR/CD-1 male mice. Consistent with previous studies, *Trichinella spiralis* infection decreased the sperm quantity and quality, and the expression levels of genes related to spermatogenesis were also suppressed including *Herc4*, *Ipo11,* and *Mrto4*. Furthermore, we found that pheromone content in male mice urine was downregulated by GC-MS analysis. Briefly, this study revealed that the downregulated urine pheromone content discouraged the female mice from mating, and the decrease in sperm quality resulted in a decline in offspring number.

## 2. Results

### 2.1. Parasitic Infection Decreased Body Weight and Induced Specific Antibody Production

**Figure 1:**
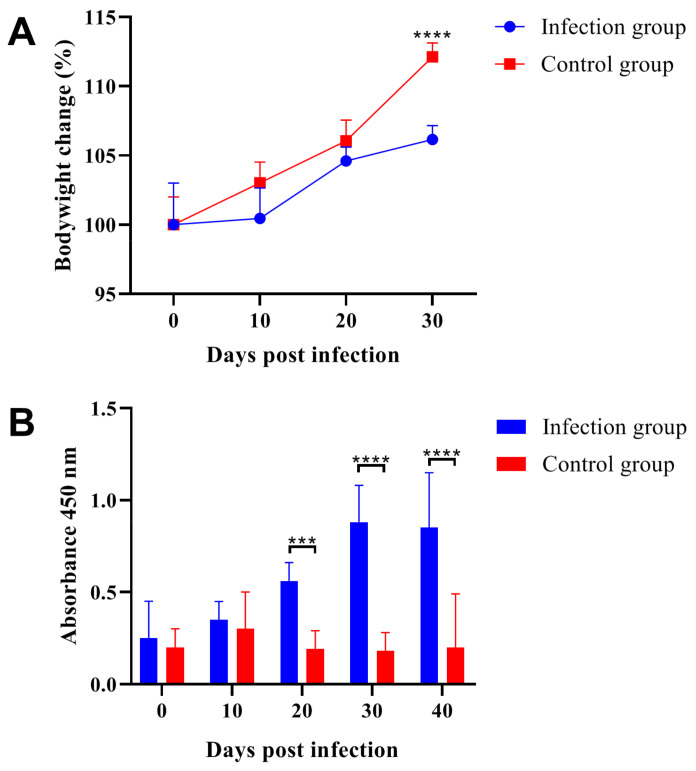
*Trichinella spiralis* infection decreased growth rate of ICR/CD-1 male mice and induced specific antibody production. (**A**) The significant differences between the infection group and control group appeared at 30 days post-infection and parasitic infection decreased host growth rate. (**B**) Parasitic infection induced specific antibody production, the results of absorbance 450 nm were determined by ELISA method and the two groups showed significant differences at 20-, 30- and 40-days post-infection. All data are presented as means ± standard deviation, Two-way ANOVA was used for significance analysis, *** *p* < 0.001, **** *p* < 0.0001.

The change in body weight in ICR/CD-1 male mice after *Trichinella spiralis* infection was presented in Figure 1A. The results indicated that 10 days after parasitic infection, the body weight in the two groups showed no significant differences. While at 30 days post-infection, the body weight of male mice in the infection group was significantly lower than that in the control group. On the other hand, we monitored the dynamic level of serum antibodies against the excretory-secretory antigen by the ELISA method (Figure 1B). The excretory-secretory antigen of *Trichinella spiralis* is a mixture that contains different proteins including transmembrane serine protease 9, serine protease, hypothetical protein T01_7775, partial P49 antigen, and partial actin 1 [27]. In some studies, the excretory-secretory antigen was usually used as a whole group [28,29]. The ELISA results indicated that *Trichinella spiralis* infection induced specific IgG production, and the absorbance showed significant differences between the two groups at 20-, 30- and 40-days post-infection. These results revealed that parasitic infection decreased the growth rate of male mice, and the parasitic infection of ICR/CD-1 male mice persisted throughout the experiment.

### 2.2. Trichinella Spiralis Infection Decreased the Attractiveness of Male Mouse Urine to Female Mice

**Figure 2:**
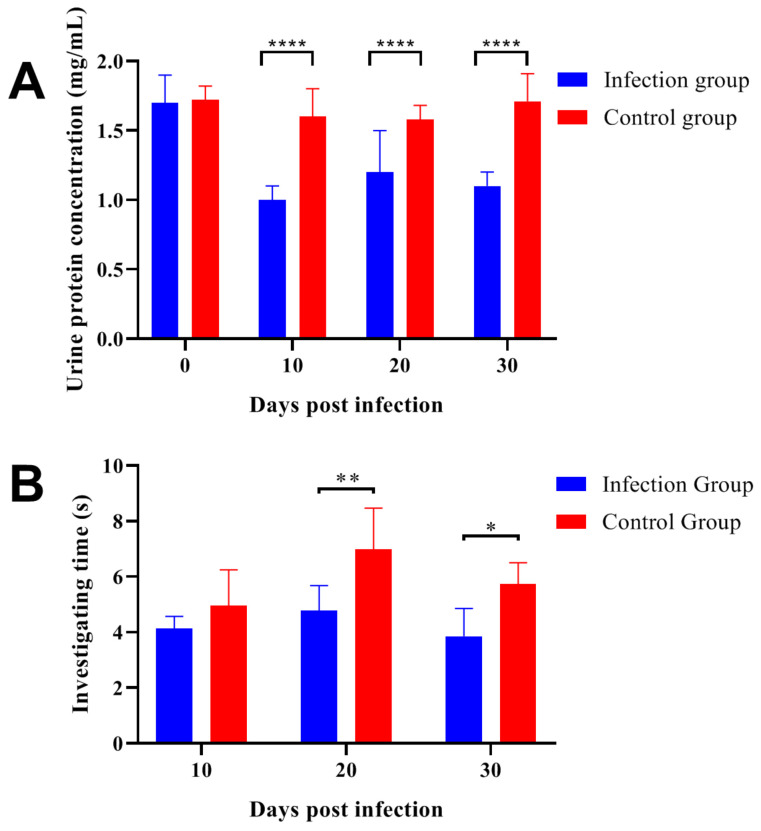
*Trichinella spiralis* infection decreased the attractiveness of male mouse urine to female mice. (**A**) At 0-, 10-, 20- and 30-days post-infection, the urine protein levels in infection group were determined and showed significant decreases when compared with the control group. (**B**) The behavioral test was performed at 10, 20 and 30 days after *Trichinella spiralis* infection, the two groups showed significant differences at 20- and 30-days post-infection. All data are presented as means ± standard deviation, Two-way ANOVA was used for significance analysis, * *p* < 0.1; ** *p* < 0.01, **** *p* < 0.0001.

The protein concentrations in urine from the infection group and control group were determined at 0-, 10-, 20- and 30-days post-infection (Figure 2A). The results indicated that the total urine protein concentration of the control group was basically stable during the experiment. While in the indicated detection time point, the urinary protein levels in the infection group were significantly lower than that in the control group. The behavioral test was performed at 10, 20, and 30 days after parasitic infection. We evaluated the attractiveness of female mice to male mice urine by recording the cumulative duration of females sniffing urine samples within 3 min (Figure 2B). The female mice spent a longer time sniffing and exploring the male mice’s urine in the control group, and the two groups showed significant differences at 20- and 30-days post-infection. These results indicated that the *Trichinella spiralis* infection reduced the urine attractiveness of male mice to females, and the poor physical aspect of male mice could discourage females from mating, which was estimated in further experiments.

### 2.3. Trichinella Spiralis Infection Significantly Decreased Pheromone Content in the Male Mice Urine

**Figure 3:**
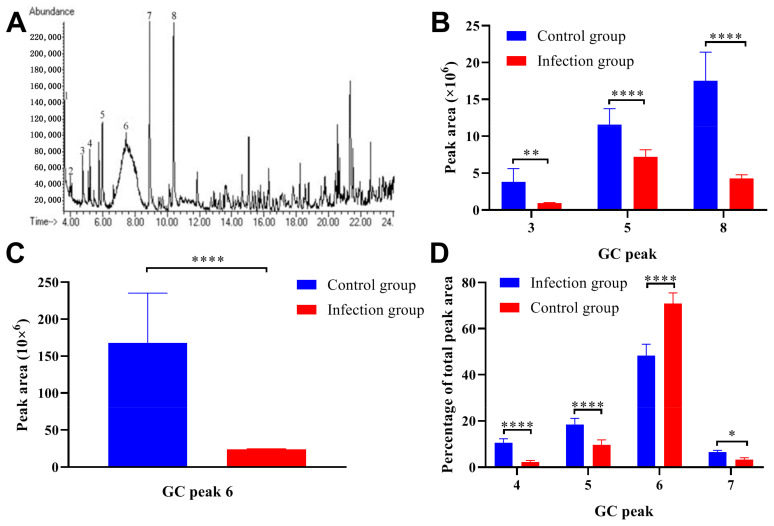
The infection of *Trichinella spiralis* decreased the pheromone content in the male mice urine. (**A**) The representative gas chromatogram from infection group was presented and the GC peaks were indicated. (**B**,**C**) The comparison of the absolute content of Z-7-tetradecen-1-ol (GC Peak 3), Dimethyl sulfone (GC Peak 5), 6-Hydroxy-6-methyl-3-heptanone (GC Peak 6), and (S)-2-sec-butyl-4,5-dihydrothiazole (GC Peak 8) between the infection group and the control group. (**D**) The comparison of the relative amount of 1-Tetradecanol (GC peak 4), Dimethyl sulfone (GC peak 5), 6-Hydroxy-6-methyl-3-heptanone (GC peak 6), and R,R-3,4-dehydro-exo-brevicomin (GC peak 7) between the infection group and control group. All data are presented as means ± standard deviation, Two-way ANOVA was used for comparison between the two groups, * *p* < 0.1, ** *p* < 0.01, **** *p* < 0.0001.

GC-MS analysis was performed to analyze the changes in pheromone content in the urine collected from the *Trichinella spiralis* infected ICR/CD-1 male mice, and the representative gas chromatogram from the infection group was presented in Figure 3A. A comparison of the pheromone content between the infection group and control group was listed in Table 1. The parasitic infection decreased the absolute content of Z-7-tetradecen-1-ol (GC Peak 3), Dimethyl sulfone (GC Peak 5), 6-Hydroxy-6-methyl-3-heptanone (GC Peak 6), and (S)-2-sec-butyl-4,5-dihydrothiazole (GC Peak 8) (Figure 3B,C). On the other hand, the relative amount of 6-Hydroxy-6-methyl-3-heptanone (GC Peak 6) was also downregulated after parasitic infection (Figure 3D).

### 2.4. Effects of Trichinella Spiralis Infection on Reproduction in Male Mice

**Figure 4:**
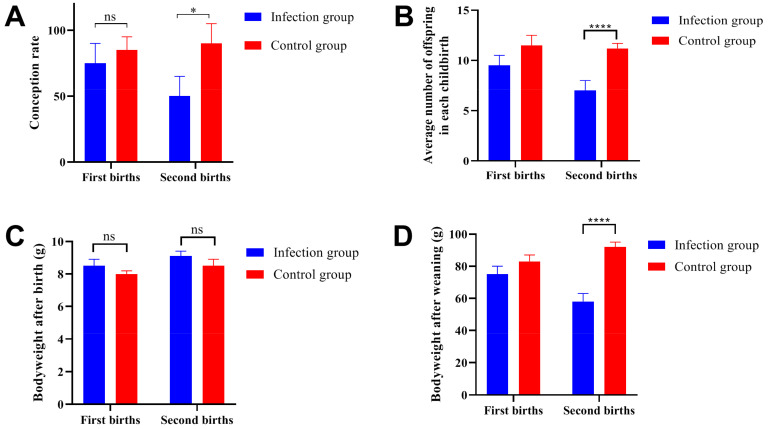
*Trichinella spiralis* infection hampered the reproductive capacity of the ICR/CD-1 male mice. (**A**) Parasitic infection decreased the conception rate of male mice. (**B**) The average number of offspring in the second births from the infection group was significantly lower than that in the control group. (**C**) The average body weight of the offspring after birth showed no significant differences between the two groups. (**D**) After weaning, the offspring body weight showed significant decreases at the second births for the infection group. All data are presented as means ± standard deviation, Two-way ANOVA was used for comparison between the infection group and the control group, * *p* < 0.1, **** *p* < 0.0001, ns non-significant.

The mating experiment was performed at 12 (first births) and 32 (second births) days post-infection, the conception rate was presented in Figure 4A. All of the male mice were used for the mating experiment and the results indicated that the conception rate of the infection group was lower than that in the control group. In the first births, the average number of offspring did not show significant differences, but in the second births, data from the infection group were significantly lower than that in the control group (Figure 4B). It should be noted that the average body weight after birth showed no significant differences between the first births and second births (Figure 4C). However, after weaning, the body weight showed significant differences in the second births between the infection group and the control group (Figure 4D). These results indicated that the *Trichinella spiralis* infection did not have a significant impact on the reproductive capacity in a short period of time (12 days post-infection), but with the extension of infection time (32 days post-infection), reproductive capacity would be significantly damaged.

### 2.5. Parasitic Infection Decreased the Sperm Quality in ICR/CD-1 Male Mice

**Figure 5:**
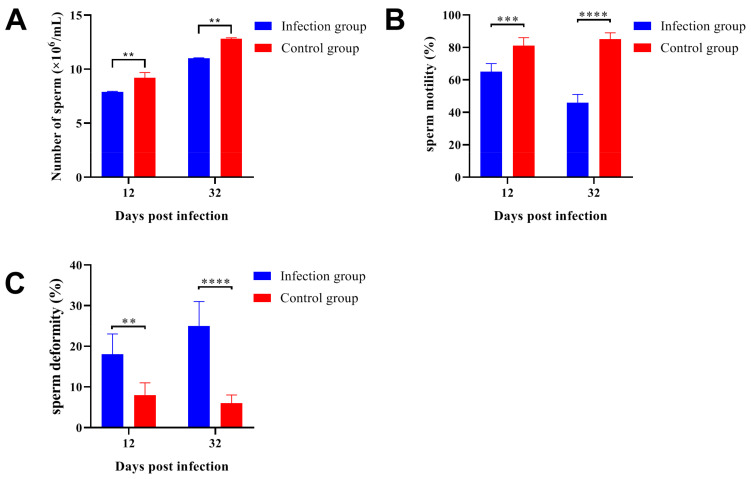
*Trichinella spiralis* infection damaged spermatogenesis and decreased the sperm quality in ICR/CD-1male mice. Sperm count (**A**) and sperm morphology (**B**) were significantly decreased after parasitic infection, and the sperm deformities (**C**) were significantly increased. It should be noted that the sperm damage caused by parasitic infection would get worse over time. All data are presented as means ± standard deviation, Two-way ANOVA was used for comparison between the infection group and the control group, ** *p* < 0.01, *** *p* < 0.001, **** *p* < 0.0001.

After *Trichinella spiralis* infection, the number of sperm separated from the infection group was significantly lower than that in the control group (Figure 5A). Furthermore, the sperm with normal morphology was also significantly decreased at 12- and 32-days post-infection (Figure 5B). On the other hand, sperm deformities (loss of hook shape, loss of head, double head, bent tail, and cytoplasmic droplet) were strongly increased in the infection group (Figure 5C). These data indicated that Trichinella spiralis infection would reduce spermatogenesis and decrease the sperm quality in infected male mice, and this damage would get worse with the extension of parasitic infection. These results could be the reason for the reproductive injury caused by *Trichinella spiralis* infection in ICR/CD-1 male mice.

### 2.6. Parasitic Infection Downregulated the Gene Expression Levels Related to Spermatogenesis 

**Figure 6:**
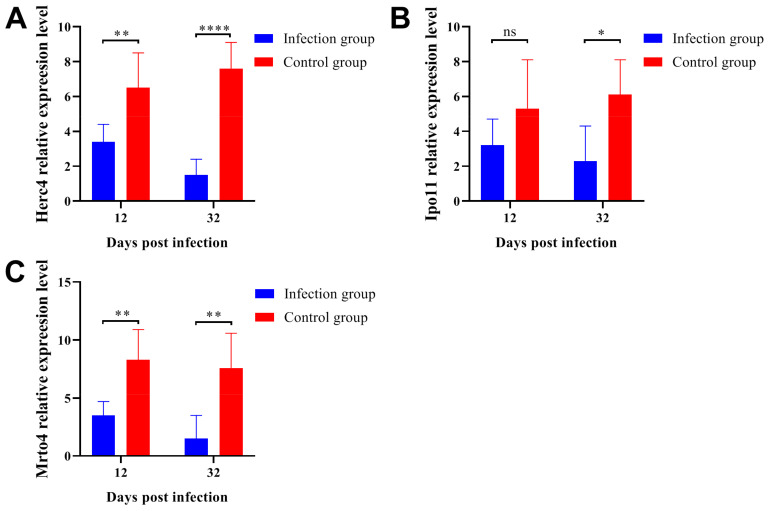
Parasitic infection downregulated the gene expression levels of *Herc4* (**A**), *Ipo11* (**B**) and *Mrto4* (**C**). The gene expression levels were determined by qRT-PCR. Each sample was tested in triplicate, and *β-actin* was used as the normalization control. All data are presented as means ± standard deviation, Two-way ANOVA was used for comparison between the two groups, * *p* < 0.1, ** *p* < 0.01, **** *p* < 0.0001, ns non-significant.

In order to assess the relationship between parasitic infection and testicular spermatogenic function, we determined the gene expression levels of *Herc4* (Figure 6A), *Ipo11* (Figure 6B), and *Mrto4* (Figure 6C) by qRT-PCR. The *Herc4*, *Mrto4,* and *Ipo11* genes are closely related to spermatogenesis and functional expression [30]. *Herc4* is an E3 ubiquitin ligase and belongs to the HERC family, the disruption of the mouse *Herc4* gene resulted in a reduction in male fertility associated with a defect in the late stages of spermiogenesis [31]. *Mrto4* can regulate the polyadenylation and deadenylation of some special mRNAs in spermatogenesis [32]. *Ipo11* is an important gene that could regulate the expression of tumor suppressor gene TSLC1/IGSF4, TSLC1/IGSF4 had been reported as an important gene to promote the differentiation and maturation of spermatogenic cells and spermatozoa [33]. These results indicated that the expression levels of *Herc4*, *Ipo11,* and *Mrto4* were significantly downregulated after parasitic infection. On the other hand, it should be noted that the gene expression levels would decrease even more as the infection lasted longer. 

## 3. Discussion

In the animal world, signals produced by one individual and received by another play an important role in controlling social behaviour and reproductive activity [34]. The urine produced by rodents provides abundant information about the host age, sex, health status, hormonal levels, and genetic background [35,36]. Pathogen infection can reduce the attractiveness of urine from infected male mice [37] and the unhealthy state can even stimulate aversion from females in mate choice [38]. In addition to *Trichinella spiralis*, studies had reported that the infection of *Dipetalonema evansi*, *Toxoplasma gondii*, *Trypanosoma vivax,* and *Neospora caninum* could affect the reproductive capacity of male rodents [1,2,3,4,5]. Consistent with the previous research results [39], the odor attraction of urine of male mice was significantly reduced after *Trichinella spiralis* infection, which was shown in female mice escaping from the urine of male mice after parasitic infection. Female individuals from the same species can distinguish from the secondary sexual characteristics such as the smell of male rats and try to avoid mating with the animals infected by pathogens [40].

After *Trichinella spiralis* infection, male mice experienced a process of weight loss. The body weight was significantly lower than that of the control group at 30 days post-infection, which indicated that the parasitic infection affected the phenotypic characteristics of the male mice. The specific antibody production against the excretory-secretory antigen was detected throughout the experimental phase. Some studies reported that after *Trichinella spiralis* infection, this specific IgG antibody could be detected in the host for a long time [41,42]. On the other hand, because of the chronic infection caused by *Trichinella spiralis* invasion, the reproductive injury may last longer and further shows the impact on population size.

The MUP family is the transporter of a variety of ligands, including pheromones, steroid hormones, retinoids, and lipids. MUPs can bind pheromones within the hydrophobic calyx of the protein structure where the hydrophobic binding sites exist for small lipophilic ligands [19]. MUP is mainly expressed in the liver with a sex-dimorphic pattern, and the expression level in males is several times higher than in females [19]. Furthermore, the expression level of MUPs can be regulated by multiple hormones including testosterone, growth hormone, and thyroxine [12]. The sexual selection of rodents to the same individuals is mainly based on the chemical signals related to MUPs. MUPs and their combined volatile pheromones can provide reliable individual information about body quality and health status [43,44,45,46].

We determined the urine protein concentration in the infection group and control group, and the results indicated that *Trichinella spiralis* infection significantly reduced the total amount of urine protein in male mice. Through the GC-MS analysis, the relative amounts of Dimethyl sulfone, Z-7-tetradecen-1-ol, 6-Hydroxy-6-methyl-3-heptanone, and (S)-2-sec-butyl-4,5-dihydrothiazole were significantly downregulated in the infection group. The downregulation of pheromone content in urine could be an important reason why male mice urine was less attractive to females, on the other hand, the poor physical aspect of male mice caused by *Trichinella spiralis* infection could discourage females from mating.

Some studies reported that the infection of *Taenia taeniaeformis* and *Heligmosomoides polygyrus* could decrease the number of offspring produced by host animals [47]. Here, we found that the total number of offspring produced by male mice infected with *Trichinella spiralis* was 31% lower than that in the healthy male mice. *Trichinella spiralis* infection downregulated the gene expression levels of *Herc4* [5,31], *Mrto4* [30], and *Ipo11* [48], genes closely related to the production and functional expression of sperm, which led to the decrease in sperm count. We also checked the sperm quality and found that sperm quality and sperm motility were significantly downregulated and sperm deformities were strongly upregulated in the experimental group. In summary, this study revealed that *Trichinella spiralis* infection could affect the reproductive capacity of male mice by decreasing urine pheromone content and sperm quality.

## 4. Materials and Methods

### 4.1. Animals

Thirty ICR/CD-1 male mice and seventy-five ICR/CD-1 female mice (6-week-old) were purchased from Weitong-Lihua Experimental Animal Company, Beijing, China. Twenty Kunming mice were also purchased from the Weitong-Lihua Experimental Animal Company and used for parasite continuous passage and separation. Both of the males and females were kept in plastic cages and the temperature was set to 22 ± 0.5 °C. Males were individually kept and females were kept in groups of three, the food and water were provided freely. All mice were virgin and in estrus, males were scrotal and females had perforated vaginas. Before the experiment, all of the mice were acclimated to the environment for 2 weeks. The protocols for animal study were approved by the Committee on the Ethics of Animal Experiments of the Institute of Zoology, Chinese Academy of Sciences (Approval number: IOZ20211010-09). Humanitarian gestures were given according to the 3R principle during the entire investigation [49].

### 4.2. Parasite Separation and Mice Infection

The *Trichinella spiralis* (genotype T1) was kept in our laboratory and preserved in Kunming mice. In brief, each mouse was infected with 350 *Trichinella spiralis* larvae. The mice were sacrificed at 35 days post-infection and the whole muscles were separated and suspended in digestion solution (1% pepsin and 1% concentrated hydrochloric acid) for three to four hours at 37 °C with a magnetic stirrer [50]. The *Trichinella spiralis* larvae were collected using the natural sedimentation method and washed three times with PBS (100 U/mL penicillin and 100 μg/mL streptomycin added) until clear. For parasitic infection in mice, thirty ICR/CD-1 male mice were divided into two groups (infection group and control group) with fifteen mice per group. The mice in the infection group were inoculated by gavage with 200 *Trichinella spiralis* larvae suspended in 200 μL of PBS, while the mice in the control group received 200 μL of PBS in the same manner. The body weight of each mouse in the control group and infection group was recorded every 10 days.

### 4.3. Preparation of Excretory-Secretory Antigen

The Kunming mice infected with *Trichinella spiralis* were sacrificed at thirty-five days post-infection, and the muscle larvae were collected with the previously published method [51]. The collected muscle larvae were washed three times with PBS buffer supplemented with 500 U/mL mycillin and transferred to RPMI 1640 medium supplemented with 500 U/mL mycillin at a density of 5000 muscle larvae/mL, then the muscle larvae were cultured in a 5% CO_2_ incubator at 37 °C for 24 h [52]. Subsequently, the culture supernatant was collected and concentrated for 100-fold on a YM-3 membrane (Amicon, Beverly, MA, USA) at 4 °C, then the culture supernatant was dialyzed in saline solution for three days and the dialysate was replaced every eight hours [53]. The protein concentration of excretory-secretory antigen was determined using the Pierce™ bicinchoninic acid protein assay kit (Thermo Scientific, Waltham, MA, USA) and stored at −80 °C until use.

### 4.4. Enzyme Linked Immunosorbent Assay (ELISA)

Blood collection of the ICR/CD-1 male mice was performed every 10 days after parasitic infection, the whole blood was placed at 4 °C for 12 h and the serum was separated by centrifugation at 4 °C for 10 min. The serum separation was performed every 10 days and all serum samples were stored at −80 °C until use. The ELISA was performed with the previously published method [54]. In brief, the excretory-secretory antigen was diluted to 1 μg/mL with the antigen coating buffer (0.05 mol/L phosphate buffer solution, pH 9.6), and a 96-well microplate was coated with 100 μL of diluted antigen at 4 °C for twelve hours. Next, the antigen solution was discarded and the microplate was washed three times with PBST buffer (supplemented with 0.5% Tween-20), and the microplate was blocked by 5% BSA in PBS at 37 °C for 2 h. The mice serum was diluted at 1:50 in antibody diluent (2% skim milk powder and 3% bovine serum albumin dissolved in PBS), and the microplate was incubated with 100 µL diluted serum for 2 h at 37 °C. After washing the microplate with PBST three times, the secondary antibody was diluted with the antibody dilution, and 100 µL of the secondary antibody was added and incubated at 37 °C for 1 h. The secondary antibody was HRP-labeled goat anti-mouse IgG (H + L) (1:2000, Beyotime, Shanghai, China). TMB single-component substrate solution (100 µL, Beyotime, Shanghai, China) was added and the reaction was stopped by the addition of 2 mol/L H_2_SO_4_. The OD450 was read with a multifunction microplate reader (Bio-Rad, Hercules, CA, USA), and the reference wavelength was set to 630 nm.

### 4.5. Mice Mating Experiment

Two mating experiments were conducted at 12 and 32 days after *Trichinella spiralis* infection in ICR/CD-1 male mice. In brief, each infected male mouse cohabited with a non-sexually experienced female mouse for 6 days for reproduction. From day 2 to day 6 after cohabitation, the appearance of a pessary in the female mice was checked every day, if the pessary appeared, the female mice would be transferred to a clean cage until childbirth. All of the female mice would be transferred to clean cages after 6 days of the first cohabitation until childbirth. All newborn mice were raised separately at 21 days after childbirth. We counted and recorded the conception rate, the number of F1 offspring, average birthweight of the newborn mice, and the body weight of the newborn mice after weaning in the two mating experiments to assess the effect of *Trichinella spiralis* infection on the reproductive ability of male mice [5].

### 4.6. Urine Collection

For urine collection, the male mice were moved to a clean cage and the cage was covered with a wire mesh (0.5 × 0.5 cm) about 2 cm from the bottom. After the mice urinated, the urine was aspirated with a disposable capillary glass tube and transferred to a centrifuge tube. The urine was collected at least 1 mL per day for a single mouse and stored at −20 °C immediately after collection. Urine collection was performed for two days from 09:00 to 16:00, the duration of a single collection for each mouse did not exceed 30 min, and any urine contaminated by feces was discarded. The protein concentration of the collected urine was determined by a Pierce™ bicinchoninic acid protein assay kit (Thermo Scientific, Waltham, MA, USA). Equal amounts of the urine collected from the same mouse over two days were mixed and stored at −20 °C for testing.

### 4.7. Behavioral Tests

The preference of female mice for male mouse urine was tested with the previously published method [55]. Before each test, a female mouse was transferred to the test room under dim light, 2 μL of urine was presented to the mouse with a disposable glass (i.d. 1.1–1.2 mm, o.d. 1.3–1.4 mm, 15 cm length) capillary through the cage cover, and one end of the capillary was sealed with plasticine. The capillary was about 1 cm away from the tip of the capillary, so the mice could not directly come into contact with the sample, and only volatiles were accessible to the subjects. Two different types of urine samples (experimental group and control group) were simultaneously presented to a female mouse, the two capillaries were lowered through the wire lid and kept approximately 2 cm apart. The attractiveness of the male mouse urine was measured by recording the cumulative duration of the behavior of female mice sniffing the urine samples within 3 min. Each mouse was chosen randomly and used only once a day, and any female mouse that did not respond to the two capillaries over the first 3 min was excluded for the day. A total of 24 ICR/CD-1 female mice were used in the behavioral tests for three consecutive days, and the urine samples collected from the experimental and control groups were all tested.

### 4.8. GC-MS Analysis

Gas chromatography-mass spectrography (GC-MS) was performed with the Agilent Technologies Network 6890 N GC system combined with a 5973 Mass Selective Detector. The GC system was equipped with an HP-5MS capillary column (30 m long × 0.25 mm i.d. × 0.25 μm film thickness). The carrier gas was helium at a flow rate of 1.0 mL/min (purity ≥ 99.999%). The temperature of the injection port was 230 °C, the initial temperature of the furnace temperature was 50 °C, and the temperature was programmed to increase to 150 °C at a rate of 5 °C/min, and then to 230 °C at a rate of 10 °C/min and maintained at this temperature for 10 min to clean the column after running the samples. For the Mass spectrometer, the electron impact ionization was set to 70 eV, and the temperature of the transfer tube was set to 280 °C, the scanning nucleo-cytoplasmic ratio (m/z) was 30–450 amu. The urine (3 μL) was injected manually in a split (10:1) mode. The mass spectra were searched using the NIST/EPA/NIH 2002 mass spectral library, and data processing was performed with Xcalibur software. Preliminary qualitative analysis of the target compound was carried out by comparing the mass spectrum of the peaks on the gas chromatogram with the mass spectrum library (NIST2002) and referring to the compound that was identified in the published papers [11,39,40,41,56,57].

Quantitative comparisons of specific compounds between the infection and control groups were performed in terms of absolute abundance and relative abundance of the compounds. The absolute abundance of a particular compound was quantified as the chromatographic peak area, and the relative abundance was the percentage of the abundance of a particular compound over the total chromatographic peak area of all identified compounds in the source sample.

### 4.9. Sperm Count and Sperm Quality Assessment

Male mice infected with *Trichinella spiralis* for 12 and 32 days were sacrificed. For sperm count, sperm from the fifth region of the left and right cauda epididymis were separated by placing the cauda epididymis in 50 mL of pre-warmed PBS buffer at 37 °C for 5 min. The sperm concentration in 1ml PBS was determined by a blood cell counting plate using an optical microscope at 100× magnification according to the standard protocol [3]. The sperm quality assessment was performed according to a previously published method [5]. In brief, the epididymises were separated immediately after the mice had been sacrificed, then the epididymises were placed in 2 mL of pre-warmed Hank’s balanced salt solution (supplemented with 0.2% bovine serum albumin, free of calcium and magnesium). The tissues were minced and incubated at 37 °C for 15 min. The sperm motility was determined with an optical microscope at 400× magnification, at least ten fields were observed, and the percentage of sperm motility was recorded. For the deformity rate calculation, the sperm suspension was air-dried and fixed in methanol, and a total of 300 sperm cells in different fields of each sample were observed and recorded.

### 4.10. Quantitative Real-Time RT-PCR (qRT-PCR)

The testicle tissues were separated immediately and frozen in liquid nitrogen after the mice were sacrificed. Total RNA was extracted from 50–100 mg of the tissues with TRIzol reagent (Invitrogen, Carlsbad, CA, USA) according to the manufacturer’s protocol. The purity of RNA was assessed from the ratio of optical densities at 260 nm and 280 nm, and the integrity was checked by 1% agarose gel electrophoresis. The first-stranded cDNA was synthesized with 2 µg of the total RNA by a PrimeScript™ II 1st Strand cDNA Synthesis Kit (TaKaRa, Beijing, China), the oligo dT primer and random 6 mers were used in the reaction system. Afterwards, the mRNA abundance of *Herc4*, *Ipo11,* and *Mrto4* were determined by a TaKaRa SYBR Green QPCR kit (TaKaRa, Beijing, China) which was performed on an ABI prism 7000 sequence detection system. The primer sequences used in qRT-PCR were listed in Table 2, and the *β-actin* was used as a normalization control to correct for any loading discrepancies. Each sample was tested in triplicate, and the data from three independent experiments were used for the final analysis.

## Figures and Tables

**Table 1:** Content comparison (Peak area and Percentage of total peak area) of the volatile compounds separated from urine by GM-MS between the infection group and control group.

GC Peak	Name	Peak Area		Percentage of Total Peak Area	
		Control Group	Infection Group	Control Group	Infection Group
1	E-β-farnesene	3.91 × 10^5^ ± 5.12 × 10^4^	2.38 × 10^5^ ± 3.11 × 10^4^	0.39 ± 0.11	0.70 ± 0.17
2	2-Heptanone	6.76 × 10^5^ ± 9.70 × 10^4^	4.88 × 10^5^ ± 5.58 × 10^4^	0.71 ± 0.24	1.33 ± 0.21
3	Z-7-tetradecen-1-ol	3.80 × 10^6^ ± 1.81 × 10^6^	9.36 × 10^5^ ± 7.68 × 10^4^	1.84 ± 0.39	2.69 ± 0.47
4	1-Tetradecanol	2.35 × 10^6^ ± 3.41 × 10^5^	4.12 × 10^6^ ± 7.28 × 10^5^	2.22 ± 0.65	10.5 ± 1.80
5	Dimethyl sulfone	1.16 × 10^7^ ± 2.13 × 10^6^	7.20 × 10^6^ ± 9.82 × 10^5^	9.62 ± 2.20	18.5 ± 2.63
6	6-Hydroxy-6-methyl-3-heptanone	1.68 × 10^8^ ± 6.74 × 10^7^	2.44 × 10^7^ ± 6.05 × 10^5^	70.9 ± 4.60	48.3 ± 4.94
7	R,R-3,4-dehydro-exo-brevicomin	6.53 × 10^6^ ± 2.64 × 10^6^	3.24 × 10^6^ ± 8.14 × 10^5^	3.26 ± 0.84	6.54 ± 0.79
8	(S)-2-sec-butyl-4,5-dihydrothiazole	1.75 × 10^7^ ± 3.90 × 10^6^	4.30 × 10^6^ ± 4.80 × 10^5^	11.00 ± 1.30	11.4 ± 1.58

**Table 2:** Primers used in this study.

Gene Name	Primer Sequence (5′-3′)	Length (bp)
*Herc4*	5′-GCCTATGGAGTGTTGGCAGA-3′	99
	5′-GACTGCATCTGTCTGGAGCA-3′	
*Mrto4*	5′-ACCTGATAGAAGAGCTTCGGA-3′	256
	5′-TCCTTCGTGCGGTTGGTAAA-3′	
*Ipo11*	5′-CACACCAGAGCTGCTTCGTA-3′	860
	5′-TTTCCATGAGGGACTGGAAG-3′	
*β-actin*	5′-TCCAGCCTTCCTTCTTGGGT-3′	111
	5′-GCACTGTGTTGGCATAGAGGT-3′	

## Data Availability

The original data presented in the study are included in the article, further inquiries can be directed to the corresponding author.
